# Our Wile(y) Friend

**Published:** 1893-10

**Authors:** 


### OUR WILE(Y) FRIEND.

"Many a shaft at random sent finds mark the archer little meant"; or "throw a stone and hear a yelp, and you may know," etc.

Our friend Dr. W. C. Wile, of the bile colored *New England Medical Journal*, has gotten his back up very unexpectedly to us, and altogether unnecessarily, about an editorial in our last, entitled the "Function of the Pharmacist." In it we were entirely disinterested, and endeavored to state, impartially, the controversy between Dr. Hammond and Parke, Davis & Co., regarding the assumed right of the latter to manufacture "certain animal extracts." We did, however, express an opinion that the medical press should not back up a physician in a proprietary interest in his discoveries.

Under the heading, "Our Red Friend," Dr. Wile indulges in an untrue and very unfair and ungenerous tirade against his "friend Daniels," putting an s to our name (we can tack a terminal s to *his* name and give it an appropriate significance), and shows a very clear bias in favor of "certain animal extracts"; in fact, so clear as to leave no doubt in our mind that he is an interested party, and owns stock in the Columbia Chemical Co. We did not know this, hence unwittingly stepped on his toes. We were surprised, more than hurt, because of the coarseness of his attack; it was such as we had not thought him capable of. His assertion, that this editorial, which has so (unintentionally) stung him, and which was neither "unfair" nor "biased," "was written with a financial leaning against one of his (my) own profession" (Dr. Hammond), as well as his unworthy suggestion in the last line, that we were actuated by mercenary motives, are *falsified* by the fact that Dr. Hammond has two and a half pages of advertisements in the TEXAS MEDICAL JOURNAL, while Parke, Davis & Co. have only one. Surely, if we had been "afraid" of losing advertising patronage for expressing our sentiments, we would have said nothing; or if it had been a matter of "financial leaning," we should have leaned toward the other party.

Dr. Wiles says, "It may be a matter of interest for our red friend to know that the *New York Medical Record* and the *New York Medical Journal* refused to publish the drug firm's, 'An Apology' in their advertising pages, and the drug firm cancelled their contract."

It may be of interest for our jaundiced friend to know that the JOURNAL does not regulate its action by that of the New York journals, and that we do not care a snap whether they did or did not do a certain thing; and further, "it may be a matter of interest" for our wiley friend of the *jaundiced* journal to know that *we* refused to print the original "On Certain Animal Extracts"—except *as* an advertisement.

Our wiley friend accuses us of "bad taste." *That* is a matter of taste, and if his standard of taste is a recent editorial, "Thanks Gentlemen Awfully," a weak and puerile kick at Parke, Davis & Co., in which he affected to thank them for advantageously advertising his journal by their merited strictures and denunciation of his course (which he could not answer), we sincerely hope that *all* our articles are "poor taste" in his estimation. He is bilious, and everything tastes bad to him. We feel assured he is ashamed of that most puerile display; but, speaking of taste, what of the boast of how much money he had made, and his ability to build a brick house? "No accounting for taste," he says. Surely not.

Wiles has gone daft on the "make," and out of the fullness of the heart the mouth speaketh.

